# On Marking Ink, for Marking Linen, &c., without the Use of a Mordant

**Published:** 1847-07

**Authors:** 


*On Marking Ink, for marking Linen, &c., without the use of a Mordant.* By Mr. Redwood.—The practice of marking linen and other similar fabrics employed as wearing apparel, or for domestic use, with a preparation of silver, commonly called *Marking Ink*, has prevailed for many years, and has now become almost universal. The preparation first introduced for this purpose consisted of a solution of nitrate of silver, thickened with gum arabic and coloured with sap green; but in using this solution it is necessary previously to apply to the article to be marked, a *preparation* or *mordant*, consisting of a solution of carbonate of soda.

The following formula has been very generally adopted in the preparation of this kind of marking ink:

| | | |
|---|---|---|
| ℞. | Carbonate of Soda | ℥ss. |
| | Distilled Water | ℥iv. |

Mix, and sign "*The preparation or Mordant.*"

| | | |
|---|---|---|
| ℞. | Nitrate of Silver | ʒj. ℈ij. |
| | Gum Arabic | ʒij. |
| | Sap green | ℈j. |
| | Distilled water | f.℥j. |

Mix and sign "*The Ink.*"

The ink made from the above, or a similar formula, which, I believe, almost every druggist through the country has been in the habit of preparing and selling, when used according to the usual instructions, produces a result which is subject to no objection that does not equally apply to any other marking ink having silver as its basis.

Within the last few years, however, the marking ink made as above, has been to a great extent superseded by the introduction of a new kind of ink, which does not require the use of a *mordant* or *preparation.* This ink appears to be generally preferred to the other; it is in one bottle, which occupies but little space, and its use is considered to be attended with less trouble and inconvenience than that of the other.

My atttention has recently been directed to this subject, as I was desirous of introducing a good formula for *marking ink, to be used as*

*a mordant*, into the new edition, now publishing, of Gray's *Supplement to the Pharmacopœia.* Several formulæ have been published in the Journals, for the preparation of this ink, but none of these have given complete satisfaction.

The following appear to be the principal requisites in this kind of ink:

1st. That it shall flow freely from the pen, and form a well defined mark without running or blotting.

2d. That it shall not require a very strong or long continued heat to be applied, by holding the article that has been written on to the fire, or passing a hot iron over it, in order to develope the black mark required.

3d. That the mark produced by it, when developed by the application of heat, or by exposure to light, shall be perfectly black.

4th. That it shall not destroy the texture of even the finest cambric.

After several experiments, I have succeeded in making a marking ink, which I think will be found to realize all the above conditions; it is thus prepared:—

| | | |
|---|---|---|
| ℞. | Nitrate of Silver | ℥j. |
| | Carbonate of Soda, crystallized, | ℥iss. |
| | Tartaric Acid | ʒij. ℈ij. |
| | Strong Liquor Ammoniæ | f.℥ij. or *q. s.* |
| | Archil | f.ʒss. |
| | White Sugar | ʒiv. |
| | Powdered Gum Arabic | ʒxij. |
| | Distilled water | *q. s.* |

Dissolve the nitrate of silver and carbonate of soda separately in distilled water: mix the solutions: collect and wash the precipitate on a filter; introduce the washed precipitate, still moist, into a Wedgewood's-ware mortar, and add to it the tartaric acid, rubbing them together until effervescence has ceased; add *liquor ammoniæ* in sufficient quantity to dissolve the tartrate of silver; then mix in the archil, white sugar, and powdered gum arabic, and add as much distilled water, if required, as will make f.℥vj. of the mixture.

It will be observed that the essential difference between this formula and those which have been already published, consists in the use of tartrate of silver, instead of nitrate of silver.—*Pharm. Journ.*

